# Supplementary material for: Complexome profiling of the Chlamydomonas psb28 mutant reveals TEF5 as an early PSII assembly factor
Source: Plant Cell. 2025 Mar 17;37(6):koaf055. doi: 10.1093/plcell/koaf055 (PMC12164590; doi:10.1093/plcell/koaf055)
Supplement: koaf055_Supplementary_Data [file koaf055_supplementary_data.zip › psb28_interactive.zip!/plots/intensity/qualityPlots/peptides/OASTL1_Cre08.g367600.t1.2.html]

### Cre08.g367600.t1.2

Trivial name: OASTL1  
SeqCoverage [median]: 17.50  
Mass: 34.77 kDa  
  
 

| Sequence | MissedCleavage | Mass [Da] | wtIntensity | muIntensity | Score |
| --- | --- | --- | --- | --- | --- |
| DKLIVTVLPSFGER | 1 | 1572.89 | 622.80 | 0.00 | 44.44 |
| INSNEAIEMAR | 0 | 1246.60 | 0.00 | 1697.14 | 100.48 |
| NLRVDLLDEVVK | 1 | 1411.80 | 487.96 | 0.00 | 42.03 |
|  |  |  | 1110.8 | 1697.1 |  |
